# The Influence of Entrepreneurial Cognition on Business Model Innovation: A Hybrid Method Based on Multiple Regressions and Machine Learning

**DOI:** 10.3389/fpsyg.2021.744237

**Published:** 2021-11-11

**Authors:** Jian Zhou, Jian Yang, Hehe Sun, Yang Liu, Xiao Liu

**Affiliations:** ^1^Department of Business Management, School of Business, Qingdao University, Qingdao, China; ^2^Department of Computer Science and Technology, Ocean University of China, Qingdao, China; ^3^The Center for Economic Research, Shandong University, Jinan, China; ^4^Cultural Industry Research Institute, Qilu University of Technology, Jinan, China

**Keywords:** entrepreneurial cognition, strategic sensitivity, knowledge diversity, business model innovation, new ventures, machine learning

## Abstract

How to achieve business model innovation (BMI) has always been a focus topic in the field of entrepreneurship. Based on cognitive theory, this study takes new ventures as the research object to build a theoretical model to explore the impact of entrepreneurial cognition on BMI choice, its intermediary mechanism, and boundary conditions. We test our framework with a sample of 242 questionnaires; the empirical research results show that entrepreneurial configuration cognition, willing cognition, and ability cognition indirectly affect the BMI of new ventures. Strategic sensitivity (SSE) mediated the relationship between entrepreneurial cognition and BMI; knowledge diversity positively moderates the relationship between SSE and BMI. Machine learning algorithm research has found three prediction models for BMI of new ventures. By constructing a theoretical analysis framework of BMI from the perspective of cognition, the results deepen the relevant research on BMI of new ventures, clarify the source of unique characteristics and capabilities of entrepreneurs, provide a new research perspective for analyzing the impact mechanism of entrepreneurial cognition on BMI, enrich the research results in the field of “situation-cognition behavior,” and further clarify the important role of knowledge in the development of new ventures.

## Introduction

With the vigorous development of information economy and the birth and application of digital technologies (such as the IoT, big data, artificial intelligence, 3D printing technology, etc.), new ventures are facing an increasingly complex and unpredictable entrepreneurial environment (Nambisan, 2017; Steininger, 2019; Secundo et al., 2021). In such a complex and ever-changing entrepreneurial environment, there are still some enterprises that stand out in the fierce market competition with their novel and unique business models and become the best in the industry. For example, Didi uses the car model under the Internet to become a unicorn in the transportation industry, JD relies on its unique B2C model to become one of the giants in the e-commerce industry, Haier keeps itself alive through the maker incubation platform. These enterprises constantly adjust their business models according to market demands, find a suitable business model for themselves, and constantly develop and grow in the fierce market competition. It can be seen that the unique business model is the key weapon for new ventures to gain profits and improve their competitive position in a highly competitive market (Bashir et al., 2020). Business model innovation (BMI) is increasingly drawing the attention of theoretical and practical fields alike.

By combing the existing research, it is found that the exploration of BMI can be divided into two main topics: one is to explore the relationship between BMI and enterprise performance, and it is found that BMI can significantly promote and enhance enterprise performance (Casadesus-Masanell and Zhu, 2013; Heider et al., 2021); the other is based on mature enterprises and discusses the antecedent variables that affect BMI from the organizational level (Bocken and Geradts, 2020; Paiola and Gebauer, 2020). Scholars mainly study from the perspectives of means oriented, constructivism, new institutionalism, and knowledge. Compared with mature enterprises, new ventures are faced with some problems, such as imperfect organizational structure and dynamic and changeable entrepreneurial situations (Liu et al., 2021; Shepherd et al., 2021). Therefore, thinking and cognition of entrepreneurs will play an important role in business model design, and entrepreneurs will determine the business model selection path of new ventures (Zhao et al., 2021). However, few scholars explore how new ventures design and form business models from the perspective of entrepreneurs; this part of the elements may be an important reason for explaining the differences in BMI of new ventures, so it is of great value to discuss and analyze the antecedents and their mechanism of BMI of new ventures from the perspective of individual entrepreneurs. When exploring the strategic decision-making and business model selection of new ventures from the perspective of individual entrepreneurs, special attention should be paid to the individual cognitive factors of entrepreneurs. This is because, when new ventures are faced with the complex and changeable entrepreneurial environment and have no complete organizational structure to rely on, how to innovate business models to gain competitive advantage often requires entrepreneurs to make strategic decisions in combination with entrepreneurial environment and previous experience (Ehret et al., 2013). Based on social cognitive theory, researchers pay full attention to the relationship among cognition, behavior, and decision-making, and combine this logical relationship with the research field of entrepreneur characteristics; the research framework of “situation-thinking behavior” has finally formed. The choice of business model of new ventures is essentially a process of entrepreneurial cognition and thinking triggered by a unique entrepreneurial environment (Martins et al., 2015; Täuscher and Abdelkafi, 2017; Abdelnaeim and El-Bassiouny, 2020).

Entrepreneurial cognition represents the processing of environmental information; how to identify opportunities and formulate corresponding strategies to make use of opportunities is the core problem to be solved in the process of growth of new ventures (George et al., 2016; Thomas et al., 2019). Social cognitive theory particularly emphasizes the influence of individual cognition and motivation on individual behavior. With the continuous development of Internet technology and increasingly transparent information, entrepreneurs need to scan and monitor various market elements and constantly search for market opportunities (Vaghely and Julien, 2010). In this process, individuals with different characteristics have different abilities to identify and grasp opportunities; entrepreneurs have the characteristics and abilities to perceive market opportunities without deliberately searching compared with non-entrepreneurs (Baum and Locke, 2004). Strategic sensitivity (SSE) refers to the sensitivity of entrepreneurs to changes in the internal and external environment (Arbussa et al., 2017), and the differences in SSE of entrepreneurs are formally caused by entrepreneurial cognition.

Therefore, based on social cognitive theory, this paper introduces the concept of SSE to explore its intermediary role in entrepreneurial cognition and BMI. On the one hand, early search of entrepreneurs for information and monitoring of various market elements will form the initial perception of entrepreneurial elements, which will affect the identification and grasp of opportunities by affecting SSE (Westhead et al., 2009; Schmitt et al., 2018). On the other hand, entrepreneurs will form a specific cognitive model in the process of processing all kinds of entrepreneurial information, which will subtly affect the SSE of entrepreneurs, and then infect and induce the BMI of new ventures (Weber and Tarba, 2014; Arbussa et al., 2017). Based on this, this paper combines the process of entrepreneurial cognition construction of entrepreneurs with the process of BMI to explore the driving factors and mechanism of the BMI of new ventures.

Besides, the influence of entrepreneurial cognition on BMI of new ventures may be influenced by other factors. Also, there may be other mechanisms and paths for entrepreneurial cognitive structure and SSE to BMI. Although the quantitative research method based on multiple regression analysis can easily confirm the causal logic relationship between variables, it is difficult to exclude the influence of various antecedents and open the “black box” of the mutual influence between variables, which makes the research logic too “constrained.” To further clarify the relationship between variables and more effectively predict the BMI behavior of new ventures, this study presents a prediction model for BMI of new ventures based on empirical research through machine learning algorithms.

We tested our framework with a sample of 242 questionnaires; the empirical research results based on MLR show that three subdimensions of entrepreneurial cognition have a significant role in promoting BMI. Different subdimensions of entrepreneurial cognition are helpful to improve the level of SSE. SSE plays a partial mediated role between entrepreneurial configuration cognition, entrepreneurial willingness cognition (Wic), entrepreneurial ability cognition (Abc), and BMI. The higher the level of knowledge diversity (Knd), the stronger the positive effect of SSE on BMI. Adopting multiple research methods is helpful to make up the deficiency of a single method and provide more credible research conclusions (Church et al., 2020). Due to the limitations of the MLR method, although the logical framework and research assumptions of this paper have been verified, the error rate of model prediction cannot be accurately estimated. After our “normal” analysis by MLR, we go to the machine learning method to further confirm the rationality of the variables selected in the article and the model constructed. The analysis of machine learning algorithm based on the decision tree shows that three prediction models can lead to BMI: the first model is SSE, entrepreneurial Wic, and entrepreneurial configuration cognition; the second model is SSE, entrepreneurial Wic, and entrepreneurial Abc; the third model is SSE, entrepreneurial Wic, entrepreneurial Abc, and Knd.

## Theory and Hypothesis

### Business Model Innovation

Timmers (1998) first put forward the concept of the business model, holding that the business model is a description of enterprise products, services, information flow structure, various participants, interests, income sources of enterprise operation, and is a process of value creation, value acquisition, and value transmission, which essentially includes two aspects: “what kind of value to create” and “how to create value.” The BMI refers to the readjustment of the existing value chain and value elements (Magretta, 2002); it is a process in which enterprises combined with internal activity elements, products, and services deliver new values to customers and meet the needs of new customers; it is an activity that changes the original value structure of enterprises (Zott et al., 2011; Foss and Saebi, 2017). Compared with incumbent enterprises, new ventures are faced with new weaknesses, such as resource shortage and highly uncertain entrepreneurial situations, which makes most new ventures more conservative and often choose to imitate the business model of existing successful enterprises. However, with the arrival of the information age represented by the Internet of Things, big data, artificial intelligence, etc., new ventures have gradually produced the power to destroy the boundaries between the original enterprises and industries, which are highlighted by the business model from closed to open, from static to dynamic (Cozzolino and Rothaermel, 2018; Müller et al., 2018). BMI has gradually become an important way for new ventures to shape competitive advantages and even subversive advantages (Foss and Saebi, 2017, 2018).

Based on clarifying the important value of BMI to new ventures, exploring its driving factors has always been an important topic in academic and practical circles. Existing research results can be summarized into two types: the first type of research starts from the internal level of enterprises and analyzes the influence of internal resources and capabilities, senior management team, organizational culture, and organizational structure on BMI. Bock et al. (2012) believe that the strategic flexibility and integration ability of enterprise resources determine whether enterprises can solve the contradiction between a business model prototype and environment. Presenza and Petruzzelli (2019) found that BMI can only be realized with the support of executives, and the support of executives is the main driving force for accelerating BMI. The second kind of research starts from the external environment of enterprises and analyzes the influence of technological innovation, market environment, and other stakeholders (such as suppliers). For example, Yang et al. (2020) found that market orientation and market opportunities played an important role in BMI, and the consumption habits and demand levels of customers in the market influenced enterprises to constantly innovate their business models. Velu and Jacob (2016) believed that market competition and business crisis pressure are important driving forces for enterprises to seek innovation opportunities, and also an important driving factor for enterprises to implement BMI.

It can be found that the existing research on BMI focuses on incumbent enterprises but pays little attention to new ventures. When exploring their influencing factors, most of them are based on internal factors or external environmental factors of enterprises, and few studies explore how new ventures design and form business models from the perspective of entrepreneurs. Given this, this paper intends to combine the social cognitive theory and cognitive perspectives of entrepreneurs to explore the driving factors of BMI of new ventures.

### Entrepreneurial Cognition and Business Model Innovation

The social cognitive theory emphasizes that human activities are determined by the interaction of individual behavior, individual cognition, and environment (Wood and Bandura, 1989). When entrepreneurs are in a highly uncertain entrepreneurial situation, their activities depend on the interaction between their cognition and individual behavior (Mitchell et al., 2002; Liñán and Chen, 2009). Compared with non-entrepreneurs, entrepreneurs have differences in characteristics, coupled with the high pressure of time, resources, and the heterogeneity and fuzziness of entrepreneurial tasks, which makes it easier for entrepreneurs to form a unique entrepreneurial thinking mode and information processing ability in entrepreneurial situations, and make corresponding strategic decisions based on this thinking mode and ability, thus helping to create a novel and unique business model that meets market demand (Sarasvathy, 2004; Presenza et al., 2020). On this basis, researchers in the field of entrepreneurial cognition mainly pay attention to the internal relationship among cognition, behavior, and decision-making, and finally form an overall logical analysis framework represented by “situation-thinking behavior” (Yacout and Vitell, 2018; Busemeyer et al., 2020). The research based on this analytical framework shows that entrepreneurial cognition is different from entrepreneurial traits, which tend to simply look for the characteristics that are different between entrepreneurs and non-entrepreneurs and apply to all situations. Entrepreneurial cognition pays more attention to the unique information processing, opportunity evaluation, thinking mode, and decision-making process of entrepreneurs under dynamic entrepreneurial situations, and can answer the reasons that lead to differences in behavior results from a deep level. Mitchell et al. (2000) put forward three different structural levels of entrepreneurial cognition based on social cognitive theory, which are configuration cognition, willingness cognition (Wic), and Abc. The three levels of entrepreneurial cognition focus on different aspects of the cognitive process, which makes the decision-making process different based on these levels of cognition. Next, this paper analyzes the influence and effect of different structural levels of entrepreneurial cognition on BMI of new ventures.

#### Entrepreneurial Configuration Cognition

Entrepreneurial configuration cognition indicates the special knowledge structure of entrepreneurs in terms of resources and assets related to the establishment and development of enterprises. Entrepreneurs can use these unique resources, assets, and knowledge structures to help achieve specific entrepreneurial tasks, including creating new enterprises, developing new entrepreneurial opportunities, and developing new business models. It is found that the cognition of entrepreneurial configuration mainly includes four dimensions, namely, the protection of entrepreneurial conception, the construction of entrepreneurial network, the acquisition of entrepreneurial knowledge, and entrepreneurial exclusive skills (Mitchell et al., 2000). It can be found that the content composition of entrepreneurial configuration cognition is intrinsically related to the BMI of new ventures (Su et al., 2020).

The awareness of the protection of entrepreneurial ideas represents the isolation mechanism that entrepreneurs have to prevent competitors from imitating, such as special patents, franchise agreements, and contracts related to entrepreneurial tasks. This isolation mechanism can allow entrepreneurs to exchange and share information of new entrepreneurial ideas without scruple, and enhance the comprehensiveness and diversity of information of new entrepreneurial ideas based on information feedback so that new entrepreneurial ideas can be fully demonstrated and discussed before commercialization, which objectively accelerates the transformation speed from new ideas to BMI (Hayter and Link, 2018). The cognition of entrepreneurial network construction represents the special and important social contract and relationship network owned and used by entrepreneurs. Since social network is considered as the main driving force of enterprise innovation, the cognition of entrepreneurial network construction can not only provide necessary information and opportunities for entrepreneurial tasks but also bring more novel ways to realize entrepreneurial value. Perry-Smith and Mannucci (2017) think that it is the key factor driving the transformation of creativity into commercialization, especially in the implementation stage of the innovative commercialization concept, which is a decisive factor in success or failure. The cognition of entrepreneurial knowledge acquisition represents the knowledge resources of production, sales, manpower, R&D, finance, etc., owned by entrepreneurs related to the completion of specific entrepreneurial tasks. Since BMI is an activity with high knowledge reserve and high resource consumption, cognition of entrepreneurs of knowledge acquisition can help accelerate the inflow of knowledge information and make a good knowledge reserve for new ventures to change their business models (Shan and Lu, 2020). Cognition of entrepreneurial exclusive skills represents exclusivity of entrepreneurs in identifying market information, enhancing enterprise competitive advantage, and promoting enterprise innovation. On the one hand, this exclusivity of skills enhances the confidence of entrepreneurs in developing unique business ideas; on the other hand, it also provides capacity support for this innovative business model development activity, which makes entrepreneurs willing and able to carry out BMI activities. Entrepreneurs will make full use of these four dimensions of entrepreneurial configuration cognition to make strategic decision-making activities, and, with the deepening of entrepreneurial activities, entrepreneurial configuration cognition of entrepreneurs, including social contract network and exclusive skills, will be continuously expanded and enriched. Based on the framework mechanism of “thinking behavior,” the expansion and enrichment of entrepreneurial configuration cognition will be reflected in the process of business model design and ultimately promote the process of business model transformation. Therefore, this study puts forward the following hypotheses:

*H1a: Entrepreneurial configuration cognition will have a significant positive impact on* BMI.

#### Entrepreneurial Willingness Cognition

Entrepreneurial Wic refers to a series of knowledge structures that entrepreneurs are willing to carry out entrepreneurial activities and promise to ensure the smooth development of entrepreneurial activities (Mitchell et al., 2000). It is found that the series of knowledge structures in the cognitive connotation of entrepreneurial intention is mainly related to the behaviors that entrepreneurs can directly implement (Krueger and Brazeal, 1994). The structures contain three dimensions; the search and capture of entrepreneurial opportunities, commitment tolerance, and the trade-off of entrepreneurial opportunities.

It can be found that the content composition of entrepreneurial Wic is closely related to new opportunity development and influences the BMI behavior of new ventures through opportunity development activities (Clausen, 2020). Specifically, the cognition of searching and capturing entrepreneurial opportunities represents the knowledge structure and skills related to seeking new situations and opportunities. This exclusive knowledge structure can help entrepreneurs enhance the openness and tendency of developing new opportunities, and transform the searched and captured entrepreneurial opportunities into entrepreneurial tasks based on constantly seeking new situations, and finally reflect the adjustment of business models. The cognition of commitment tolerance represents that entrepreneurs are willing to put their entrepreneurial commitment into practice and bear the risks and responsibilities caused by strategic decision-making and BMI. Research shows that BMI originates from the schema mutation of valuable information stored in the minds of entrepreneurs. In this process, the cognitive inertia of entrepreneurs is an important reason for their failure to take business model change actions when faced with sudden environmental changes (Dewald and Bowen, 2010). Commitment tolerance can help overcome this entrepreneurial cognitive inertia and promote cognitive schema mutation. The cognition of the trade-off of entrepreneurial opportunities represents the belief that entrepreneurs motivate themselves to complete tasks constantly and firmly believe that losing entrepreneurial opportunities is worse than trying and failing. This belief makes entrepreneurs pay more attention to the exploitability of opportunities rather than the risk of opportunities when weighing the information of entrepreneurial opportunities, and further influences their reasoning logic, thus promoting entrepreneurs to try and innovate constantly. Also, existing research found that entrepreneurs have stronger opportunities to search and balance and promise tolerance than non-entrepreneurs, which means that entrepreneurs have stronger ability of information integration and information deconstruction, and can identify entrepreneurial opportunities in numerous information resources (Haynie et al., 2012; Wood and McKelvie, 2015). Therefore, BMI can be carried out more effectively, and enterprises can gain unique competitive advantages. Therefore, this study puts forward the following hypothesis:

*H1b: Entrepreneurial* Wic *will have a significant positive impact on* BMI.

#### Entrepreneurial Ability Cognition

Entrepreneurial Abc refers to the knowledge structure that entrepreneurs can mobilize skills, knowledge, values, and attitudes to support the creation and growth of new enterprises (Mitchell et al., 2000). Seawright et al. (2008) found that Abc mainly includes four dimensions, namely, entrepreneurial experience involvement, entrepreneurial task diagnosis, entrepreneurial situational knowledge, and ability and opportunity matching.

It can be found that consistent with the cognition of entrepreneurial willingness, the entrepreneurial Abc can also improve the innovation level of the business model by continuously exploring entrepreneurial opportunities. Specifically, the cognition of entrepreneurial experience involvement represents the knowledge structure of entrepreneurship, industry, and functional management acquired by entrepreneurs from their previous experiences. This kind of cognition can help entrepreneurs improve their investment in the creation and development of new ventures, and the higher the investment, the more they will promote enterprise innovation and keep competitive advantage. The cognition of entrepreneurial task diagnosis represents the knowledge structure that entrepreneurs have to evaluate the ability and possibility of BMI, which helps entrepreneurs to evaluate and diagnose which ideas are feasible in real time, and ensure the smooth implementation of BMI by evaluating various systematic factors in the process of BMI. The cognition of entrepreneurial situation knowledge represents the knowledge structure that entrepreneurs learn from experience and lessons in the process of BMI, and make a rational application in the face of new entrepreneurial situations and business model change. The complexity and the uncertainty of entrepreneurial situations require entrepreneurs to have a dynamic situation cognition system and can adopt different coping strategies by interpreting different entrepreneurial situations, thus realizing BMI in complex entrepreneurial situations.

The cognition of capability-opportunity matching represents that entrepreneurs discover market demand in time and reshape enterprise value chain by recombining products and services to meet the new needs of customers and create knowledge structure of value. BMI is, essentially, an activity that changes the original value structure of enterprises (Zott et al., 2011; Foss and Saebi, 2017). The activity of reshaping value structure needs to make an agile response while discovering market opportunities, and capability-opportunity matching meets the core elements required by this agile response. Based on the above analysis, the entrepreneurial Abc can give full play to the unique potential of entrepreneurs, help entrepreneurs identify key information of entrepreneurship, diagnose strategies in BMI, acquire entrepreneurial (frustration) experience and analyze entrepreneurial market support, and urge entrepreneurs to make BMI decisions beneficial to entrepreneurial growth (Urban, 2008). Therefore, this study puts forward the following hypothesis:

*H1c: Entrepreneurial* Abc *will have a significant positive impact on* BMI.

### Entrepreneurial Cognition and Strategic Sensitivity

SSE refers to the sensitivity of entrepreneurs to changes in the internal and external environment and emphasizes the continuous monitoring of various elements, which is a kind of character and ability combined with discovery opportunities (Arbussa et al., 2017). Under the entrepreneurial situation, the attitude and motivation of entrepreneurs have a profound impact on the level of SSE. The protection and commitment, knowledge, values, and attitude of entrepreneurs in the process of entrepreneurial activities are important components of entrepreneurial cognition. Due to the imperfection of organizational structure and the decision-making process of new ventures, entrepreneurs occupy a dominant position in the process of strategic decision-making and new opportunity development. As a kind of character and ability combined with discovery opportunities, when entrepreneurs attach importance to cultivating their potential, SSE is easier to play its key value in strategic decision-making and BMI. The three levels of knowledge structure contained in entrepreneurial cognition provide sufficient resource support for SSE. Cognition of commitment and attitude of entrepreneurs is helpful to enhance the attention and understanding of new strategic ideas and promote the promotion of SSE (Mitchell et al., 2000). In the external environment of entrepreneurship, entrepreneurs need to monitor the changes in market environment. Individuals perceive the changing trend of external market information and monitor and analyze this information according to the formed cognitive model to discover market opportunities. In the highly uncertain entrepreneurial environment, from the perspective of entrepreneurial cognition, entrepreneurs can better perceive internal and external information, discover market opportunities, and make better strategic decisions.

The concept protection dimension in the cognition of entrepreneurial configuration builds an isolation mechanism to prevent competitors from imitating so that entrepreneurs can develop and discuss new ideas without scruple, which builds a good atmosphere for SSE. Also, the cognitive dimension of entrepreneurial knowledge acquisition in entrepreneurial configuration cognition urges entrepreneurs to continuously own, acquire and use special financial, human, and other assets or resources necessary for entrepreneurial growth, and use these resources to discover and create market opportunities, and then make strategic decisions. Therefore, this study puts forward the following hypothesis:

*H2a: Entrepreneurial configuration cognition will have a significant positive impact on SSE.*

The dimension of commitment tolerance in the cognition of entrepreneurial willingness promotes the risk preference of entrepreneurs and is more inclined to constantly seek to develop new ideas. Also, the dimension of commitment tolerance in the cognition of entrepreneurial willingness helps entrepreneurs persist in searching for entrepreneurial opportunities that are not perceived or even denied by corporate stakeholders. Therefore, this study puts forward the following hypothesis:

*H2b: Entrepreneurial Wic will have a significant positive impact on SSE.*

The entrepreneurial experience involvement dimension in the cognition of entrepreneurial ability enables entrepreneurs to improve their knowledge input based on learning the previously accumulated knowledge structure. The ability and opportunity matching dimension in the cognition of entrepreneurial ability can help entrepreneurs identify information, diagnose business strategies, and obtain business support (Zott et al., 2011; Wood and McKelvie, 2015). Also, starting from the internal behavior of new ventures, it can be found that entrepreneurs need to rationally allocate internal resources to help enterprises make better strategic decisions while perceiving and grasping opportunities. Therefore, this study puts forward the following hypothesis:

*H2c: Entrepreneurial Abc will have a significant positive impact on SSE.*

### Strategic Sensitivity and Business Model Innovation

In essence, BMI is a kind of logic that uses new ideas and methods to construct new creations and gain value (Casadesus-Masanell and Zhu, 2013). BMI requires entrepreneurs to have the willingness to pursue innovation, business model change, and accept this willingness from attitude and motivation. It also needs entrepreneurs to have the ability to make changes and give full play to their advantages in organizational resources and opportunity development. SSE can support BMI of new ventures from perception and resources (Doz and Kosonen, 2010). Brown et al. (2019) found that SSE mainly includes three core connotations: (1) sensing the information of changes in internal and external market environment; (2) monitoring each factor and connecting the perceived new market change information with previous experience; (3) searching for feasible market opportunities and making strategic decisions.

In terms of perception, the change information of external environment stimulates the willingness of entrepreneurs to innovate in the business model. When entrepreneurs sensitively perceive the change of external market environment, they will connect this change with previous experience to form a new perception schema, which will help entrepreneurs to explore new ideas more actively (Tang et al., 2012; Gill et al., 2021). Also, continuous cognitive monitoring of entrepreneurs of external factors can also help weaken cognitive bias, guide entrepreneurs to correct their ideas, and show behaviors conducive to BMI.

In terms of resources, it can be found from the connotation of SSE that it plays a key role in the process of identifying and developing entrepreneurial opportunities (Clauss et al., 2019). On the one hand, the monitoring of external factors by SSE enables entrepreneurs to have the ability to perceive market demand or underutilize resources in a highly uncertain entrepreneurial environment, including continuous collection and processing of information, conceptual matching between specific market demand and specific resources. On the other hand, based on the theory of entrepreneurial opportunity, it is found that SSE will enable entrepreneurs to make reasonable decisions and design business models that meet market demand (Gaglio and Katz, 2001). Through SSE, the ability to evaluate and utilize opportunities can be enhanced. Under the entrepreneurial situation of “new weakness,” sensitive entrepreneurs can make full use of the resources at hand, creatively utilize the resources neglected by others, combine different opportunity development models, and value creation models to realize BMI through continuous opportunity development. Therefore, this study puts forward the following hypothesis:

*H3:* SSE *will have a significant positive impact on* BMI.

Based on the above analysis, this study holds that the level of entrepreneurial cognition can significantly affect the BMI of new ventures, and SSE mediates the relationship between entrepreneurial cognition and BMI. Specifically, the cognition of entrepreneurial allocation urges entrepreneurs to continuously own, acquire, and use special financial, human, and other assets or resources necessary for entrepreneurial growth, and use these resources to discover and create market opportunities, thus enhancing the level of SSE. Guided by a perceptual schema, SSE is constantly linked with previous experience, guiding entrepreneurs to revise their own ideas, weakening cognitive bias, and improving the efficiency of transforming into BMI. Therefore, this study puts forward the following hypothesis:

*H4a: SSE mediated the relationship between entrepreneurial configuration cognition and BMI.*

The awareness of entrepreneurial willingness helps entrepreneurs persist in searching for entrepreneurial opportunities that are not perceived or even denied by enterprise stakeholders, which improves the attention and understanding of surrounding strategic information, and, at the same time, provides sufficient resource support for subsequent strategic decisions. At the same time, by combining SSE with opportunity evaluation and opportunity development, entrepreneurs have the ability to perceive market demand and underutilize resources, and can make full use of resources at hand and creatively utilize resources neglected by others, thus creating favorable conditions for BMI. Therefore, this study puts forward the following hypothesis:

*H4b: SSE mediated the relationship between entrepreneurial Wic and BMI.*

The ability-opportunity matching dimension in the cognition of entrepreneurial ability can help entrepreneurs identify information, diagnose business strategies, and obtain business support, thus enhancing the effectiveness of SSE. At the same time, SSE can transform the previous entrepreneurial ability of entrepreneurs into different dimensions of BMI, and play the role of a bridge and a link between ability and BMI. Therefore, this study puts forward the following hypothesis:

*H4c: SSE mediated the relationship between entrepreneurial Abc and BMI.*

### The Moderating Effect of Knowledge Diversity

Strategic sensitivity emphasizes the continuous monitoring of various elements (Arbussa et al., 2017), which is a dynamic monitoring process and will be affected by many elements. The research of “environment-cognition behavior” framework based on social cognitive theory found that knowledge is actionable information, a unity of belief, experience, and information, a dynamic process rooted in interaction and time (Leonard and Sensiper, 1998). Therefore, the diversity of knowledge will affect the judgment of entrepreneurs of SSE and lead to different conversion efficiencies of SSE, which will eventually show the difference of the BMI level.

Knowledge diversity refers to the diversity of knowledge resources, such as information, knowledge, skills, and experience owned by enterprises (Vegt and Bunderson, 2005). Research shows that Knd plays three roles in the transformation from SSE to BMI of new ventures: First, the entrepreneurial situation information is complex and changeable; entrepreneurs are difficult to make choices in the face of massive information. The higher degree of Knd means that entrepreneurs can process entrepreneurial opportunity information more efficiently and help enterprises make strategic decisions. Secondly, Knd can be specifically divided into two aspects: external knowledge capture diversity and internal knowledge innovation diversity. Among them, external knowledge capture diversity can enable new ventures to acquire new technologies and opportunities in time. The diversity of internal knowledge innovation can promote the internal team of new ventures to learn new knowledge, thus promoting enterprises to transform entrepreneurial opportunities into BMI. Finally, scholars in the field of knowledge resources foundation believe that the difference in the number and structure of knowledge resources owned by enterprises will promote the innovation of enterprises (Vasudeva and Anand, 2011). This mainly reflected that a large amount of knowledge accumulation can promote the innovation of enterprises, and knowledge resources with different structures can help new ventures to create new products and services that better meet customer needs (Sapienza et al., 2005). Therefore, this study puts forward the following hypothesis:

*H5: Knd positively moderates the relationship between SSE and BMI, such that the higher the level of Knd, the stronger the positive effect between entrepreneurial SSE and BMI.*

**FIGURE 1:**
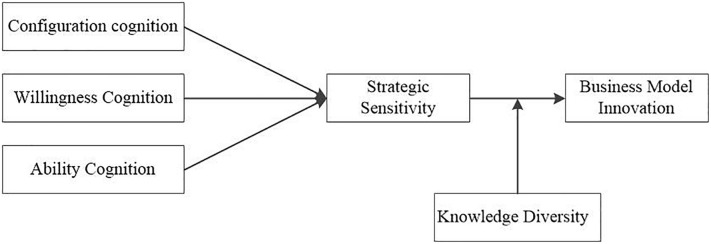
Research model.

Figure 1 depicts the conceptual model in this study.

## Materials and Methods

### Sample

In this paper, the selected sample respondents are the entrepreneurs who are in progress or have entrepreneurial experience in the recent 3 years, and the data are collected by a questionnaire. From May to June 2020, the research team distributed and collected questionnaires in eastern provinces and cities in China with active entrepreneurship, such as Beijing, Tianjin, Shandong, Jiangsu, Zhejiang, Fujian, and Guangdong. During the period, the research team distributed about 500 questionnaires, of which about 150 were distributed in paper, and 105 were recovered. The electronic version of the questionnaire was about 350, and 148 were recovered. After excluding invalid questionnaires with a response rate of 90% or less, descriptive statistics of sample characteristics are shown in Table 1.

**TABLE 1:** Characteristics of the respondents (*n* = 242).

Characteristics of the respondents		Number of responses	Percentage %
Gender	Male	168	69.4
	Female	74	30.6
Education	Junior school or below	3	1.2
	Senior high school	4	1.7
	Vocational school	19	7.9
	University	170	70.2
	Master’s/Ph.D.	46	19.0
Age	25 or younger	113	46.7
	31–40	116	47.9
	41–50	10	4.1
	51 or older	3	1.2
Founding time	Under 1 year	22	9.1
	2–3 years	92	38.0
	4–5 years	54	22.3
	6–8 years	74	30.6
Number of employees	Under 10	27	11.2
	10–50	86	35.5
	51–100	90	37.2
	More than 100	39	16.1

### Measures

In this study, all items were measured on a seven-point Likert scale, ranging from 1 (strongly disagree) to 7 (strongly agree). The survey was administered in Chinese. The measures used in this study were originally designed in English and were then translated into Chinese with the assistance of two Ph.D. candidates. To ensure equivalence of meaning (Brislin, 1970), these measures were back translated into English by another Ph.D. candidate. Two management professors then checked the instrument and made modifications to correct for discrepancies. To ensure content validity, we consulted three entrepreneurs to ask for their advice and modified according to their comments.

Entrepreneurial cognition was operationalized with three subdimensions: entrepreneurial configuration cognition, entrepreneurial Wic, and entrepreneurial Abc. A nine-item scale was obtained from Mitchell et al. (2000) to measure entrepreneurial cognition. Among them, entrepreneurial configuration cognition includes three items, such as conception protection, network construction, and resource ownership; entrepreneurial Wic includes three items, such as opportunity capture, commitment limit, and opportunity balance; entrepreneurial Abc includes three items, such as opportunity/ability matching, diagnostic ability, and situational knowledge.

Strategic sensitivity was measured with Doz and Kosonen (2010) four items, which mainly include constantly exploring and using new concepts, constantly listening to external voices, and constantly having frank, open, and fruitful dialogs around strategic issues.

Knd was measured with Kohli et al. (1993) and Jansen et al. (2005) four items, mainly including the Knd of new ventures in competitors, emerging technologies, customers, and suppliers.

BMI was measured with Luo et al. (2018) 16 items, mainly including pioneering BMI and perfect BMI.

### Common Method Bias

To reduce the problem of common method biases, on the one hand, in the process of collecting data, the research group collects data separately from the explained variables as far as possible. On the other hand, this paper carries out non-rotated principal component analysis on all items of the four variables: entrepreneurial cognition, SSE, Knd, and BMI (Podsakoff and Organ, 1986). The results show that the non-rotated first factor can explain 26.68% of the variance, and the cumulative one can explain 58.80% of the variance. The variance explained by the first factor does not reach half of the total explained variance, so it can be considered that there is no serious problem of common method biases in this study.

### Model Design

Combined with theoretical deduction and research hypothesis, the regression model established in this paper is as follows:


(1) $$\begin{array}{c} \mathrm{SSE}=\alpha_{1}+\alpha_{2}\mathrm{Arc}+\alpha_{3}\mathrm{Wic}+\alpha_{4}\mathrm{Abc}\\ +\alpha_{i}{\mathrm{Control}}_{\mathrm{gender},\mathrm{age},\mathrm{major},\mathrm{years},\mathrm{number},{\mathrm{size}}^{+\varepsilon }} \end{array}$$

(2) $$\begin{array}{c} \mathrm{BMI}=\alpha_{1}^{\prime }+\alpha_{2}^{\prime }\mathrm{Arc}+\alpha_{3}^{\prime }\mathrm{Wic}+\alpha_{4}^{\prime }+\alpha_{5}\mathrm{Sse}\\ +\alpha_{i}^{\prime }{\mathrm{Control}}_{\mathrm{gender},\mathrm{age},\mathrm{major},\mathrm{years},\mathrm{number},{\mathrm{size}}^{+\varepsilon }} \end{array}$$

(3) $$\begin{array}{c} \mathrm{BMI}=\alpha_{i}^{{}^{\prime \prime }}+\alpha_{i}^{{}^{\prime \prime }}\mathrm{Arc}+\alpha_{i}^{{}^{\prime \prime }}\mathrm{Wic}+\alpha_{i}^{{}^{\prime \prime }}\mathrm{Abc}+\alpha_{i}^{\prime }\mathrm{Sse}+\alpha_{i}\mathrm{Knd}\\ +\alpha_{i}\mathrm{Knd}\times \mathrm{Sse}+\alpha_{i}^{{}^{\prime \prime }}{\mathrm{Control}}_{\mathrm{gender},\mathrm{age},\mathrm{major},\mathrm{years},\mathrm{number},{\mathrm{size}}^{+\varepsilon }} \end{array}$$

Among them, the explained variables are BMI (BMI) and SSE. The main explanatory variables are entrepreneurial configuration cognition (Arc), entrepreneurial Wic, entrepreneurial Abc, Knd, and the interactive item (Knd × Sse).

## Results

### Confirmatory Factor Analysis

To test the discrimination validity between the key variables involved in this paper, such as “entrepreneurial configuration cognition,” “entrepreneurial willingness cognition,” “entrepreneurial Abc,” “strategic sensitivity,” “Knd,” and “BMI”; the structural equation model (AMOS 22.0) is used for confirmatory factor analysis. As the number of items in the core variable “BMI” involved in this paper is too large, resulting in the actual sample data does not meet the adequate standard. Therefore, the measurement items of BMI are factor packaged to minimize the deviation of parameter estimation. The specific analysis results are shown in Table 2.

**TABLE 2:** Results of confirmatory factor analysis for the measures of the variables studied.

	*χ2*	*Df*	*RMSEA*	*RMR*	*CFI*	*TLI*	*SRMR*
Null model^*a*^	199.99	89	0.07	0.06	0.91	0.88	0.06
Six-factor model	200.00	89	0.07	0.05	0.92	0.90	0.05
Five-factor model^*b*^	229.96	94	0.08	0.06	0.90	0.87	0.06
Four-factor model^*c*^	212.19	98	0.07	0.06	0.91	0.89	0.06
Three-factor model^*d*^	237.92	101	0.08	0.06	0.89	0.87	0.06
Two-factor model^*e*^	243.86	0.3	0.08	0.06	0.89	0.87	0.06
One-factor model^*f*^	257.03	104	0.08	0.06	0.88	0.86	0.06

*n = 242.*

*^a^In the null model, there is no relation between all measurements.*

*^b^Strategic sensitivity and knowledge diversity merged as a potential factor.*

*^c^Three subdimensions of entrepreneurial cognition merged as a potential factor.*

*^d^Three subdimensions of entrepreneurial cognition and knowledge diversity merged as a potential factor.*

*^e^Three subdimensions of entrepreneurial cognition, strategic sensitivity, and knowledge diversity merged as a potential factor.*

*^f^All measurements merged as a potential factor.*

The transverse analysis shows that all the fitting indexes meet the corresponding standards [χ2 (89)/df = 2.25, RMSEA = 0.07, CFI = 0.92, TLI = 0.90, SRMR = 0.05]. The longitudinal analysis shows that the fitting indexes of the six-factor model constructed in this paper are better than those of other factor models, which shows that the model constructed in this paper has good construction validity and good discrimination validity among variables.

### Descriptive Statistics

Table 3 lists the mean, standard deviation, and correlation coefficient of the main variables involved in this paper. From the results, it can be seen that the entrepreneurial configuration cognition (*r* = 0.66, *p* < 0.01), entrepreneurial willingness cognition (*r* = 0.60, *p* < 0.01), and entrepreneurial Abc (*r* = 0.51, *p* < 0.01) are significantly positively correlated with BMI. The entrepreneurial configuration cognition (*r* = 0.46, *p* < 0.01), entrepreneurial willingness cognition (*r* = 0.42, *p* < 0.01), and entrepreneurial Abc (*r* = 0.43, *p* < 0.01) are significantly positively correlated with strategic sensitivity. Strategic sensitivity is positively correlated with BMI (*r* = 0.62, *p* < 0.01). The results are consistent with the direction of assumptions 1–3 in this paper. Besides, it can be seen from the results in Table 3 that Cronbach’s Alpha reliability coefficients of the core variables in this paper are all greater than 0.65.

**TABLE 3:** Means, standard deviations, and correlations.

变量	1	2	3	4	5	6	7	8	9	10	11	12
(1) Gender	1											
(2) Age	0.06	1										
(3) Major	0.06	–0.08	1									
(4) Years	0.00	0.65**	–0.04	1								
(5) Number of jobs	–0.08	0.17**	0.08	0.16**	1							
(6) Scale	0.13	0.18**	−0.19**	0.25**	0.04	1						
(7) Arc	0.06	0.11	–0.13	0.11	–0.12	0.16**	1					
(8) Wic	–0.01	0.07	−0.17**	0.01	–0.08	0.12	0.51**	1				
(9) Abc	–0.03	0.18**	−0.23**	0.17**	–0.09	0.11	0.52**	0.45**	1			
(10) SSE	0.08	0.13	−0.17**	0.10	–0.08	0.06	0.46**	0.42**	0.43**	1		
(11) Knd	–0.07	0.08	–0.09	0.12	–0.10	0.10	0.35**	0.37**	0.40**	0.32**	1	
(12) BMI	0.00	0.17**	−0.15*	0.19**	–0.12	0.09	0.66**	0.60**	0.51**	0.62**	0.42**	1
Cronbach’s alpha	*N/A*	*N/A*	*N/A*	*N/A*	*N/A*	*N/A*	0.77	0.77	0.89	0.66	0.89	0.85
Mean	0.31	2.60	3.36	6.10	2.05	3.63	4.99	4.85	4.90	5.38	4.73	5.23
SD	0.46	0.64	1.10	3.83	1.32	1.70	0.96	1.02	1.00	0.94	0.87	0.75

*n = 242.*

*Significant at the p < 0.05 (**p < 0.01) level.

*N/A indicates not suitable for analysis.*

### Hypothesis Testing

**FIGURE 2:**
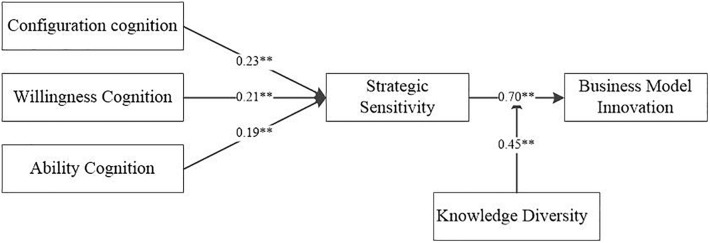
Hypothetical test results. **Significant at the *p* < 0.01 level.

In this paper, hierarchical regression is used to test Hypotheses 1–4, and nine regression test models are constructed. The specific regression test results are shown in Table 4 and Figure 2. Model 1 and Model 3 are the benchmark models, which respectively represent the influence of the six control variables in this paper on strategic sensitivity and BMI; Model 2 shows that entrepreneurship configuration cognition (β = 0.23, *p* < 0.01), entrepreneurship willingness cognition (β = 0.21, *p* < 0.01), and entrepreneurship Abc (β = 0.19, *p* < 0.01) have significant positive effects on strategic sensitivity, and Hypotheses 2a-2c are supported; Model 4 shows that entrepreneurship configuration cognition (β = 0.41, *p* < 0.01), entrepreneurship willingness cognition (β = 0.34, *p* < 0.01), and entrepreneurship Abc (β = 0.11, *p* < 0.05) have significant positive effects on BMI, and Hypotheses 1a–1c are supported; Model 5 shows that strategic sensitivity has a significant positive impact on BMI (β = 0.70, *p* < 0.01), and Hypothesis 3 is supported; Model 6 shows that, when entrepreneurial cognition and strategic sensitivity are simultaneously analyzed by regression analysis, the impact of entrepreneurial configuration cognition and entrepreneurial willingness cognition on BMI is significantly reduced, while the impact of entrepreneurial Abc on BMI becomes insignificant, which indicates that strategic sensitivity plays a partial mediating effect, and Hypothesis 4 (4a–4c) is also supported.

**TABLE 4:** Results of hypotheses testing.

Effects	Outcome: SSE		Outcome: BMI						
	M_1_	M_2_	M_3_	M_4_	M_5_	M_6_	M_7_	M_8_	M_9_
**Control variables**									
Gender	0.08 (1.27)	0.08 (1.46)	0.01 (0.20)	0.01 (0.13)	-0.05 (–0.99)	–0.03 (–0.90)	0.04 (0.65)	–0.03 (–0.78)	–0.04 (–0.95)
Age	0.08 (0.90)	0.04 (0.46)	0.05 (0.64)	0.00 (0.03)	0.00 (0.00)	–0.01 (–0.30)	0.06 (0.75)	–0.01 (–0.23)	0.00 (0.06)
Major	–0.18^∗∗^	–0.09	–0.16^∗^	–0.04	–0.03	0.00	–0.13	0.00	0.00
	(–2.74)	(–1.45)	(–2.46)	(–0.91)	(–0.71)	(–0.06)	(–2.23)	(–0.11)	(0.00)
Years	0.07 (0.76)	0.04 (0.48)	0.18^∗^ (2.08)	0.15^∗^ (2.52)	0.13 (2.18)	0.13 (2.76)	0.13 (1.60)	0.13 (2.62)	0.12^∗^ (2.37)
Number of jobs	–0.08	–0.01	–0.14^∗^	–0.05	–0.09	–0.05	–0.10	–0.04	–0.04
	(–1.24)	(–0.25)	(–2.23)	(–1.15)	(–1.90)	(–1.24)	(–1.65)	(–1.14)	(–1.19)
Scale	–0.04	–0.08	–0.02	–0.09	0.01	–0.05	–0.04	–0.05	–0.05
	(–0.54)	(–1.32)	(–0.26)	(–1.83)	(0.18)	(–1.30)	(–0.64)	(–1.37)	(–1.40)
**Independent variables**									
Arc		0.23^∗∗^ (3.24)		0.41^∗∗^ (7.47)		0.31^∗∗^ (6.72)		0.30^∗∗^ (6.60)	0.30^∗∗^ (6.58)
Wic		0.21^∗∗^ (3.10)		0.34^∗∗^ (6.43)		0.24^∗∗^ (5.57)		0.23^∗∗^ (5.26)	0.23^∗∗^ (5.23)
Abc		0.19^∗∗^		0.11^∗^		0.02		0.01	0.00
		(02.80)		(2.05)		(0.47)		(0.18)	(0.03)
**Mediator**									
SSE					0.70^∗∗^	0.47^∗∗^		0.46^∗∗^	0.47^∗∗^
					(15.33)	(11.08)		(10.88)	(10.95)
**Moderator**									
Knd							0.38^∗∗^ (6.33)	0.06 (1.57)	0.06 (1.56)
**Interaction**									
Knd × SSE									0.45^∗∗^ (12.31)
R^2^	0.06	0.30	0.09	0.57	0.55	0.72	0.22	0.72	0.73
Δ*R*^2^	0.06	0.24	0.09	0.48	0.46	0.15	0.13	0.00	0.01
*F*	2.49	10.82^∗∗^	3.68	84.15^∗∗^	234.89^∗∗^	122.76^∗∗^	40.04^∗∗^	2.46	15.15^∗∗^
Δ*F*	2.49	25.88^∗∗^	3.68	33.14^∗∗^	39.88^∗∗^	57.96^∗∗^	9.40^∗∗^	53.25^∗∗^	49.05^∗∗^

*n = 242.*

*Significant at the p < 0.05 (**p < 0.01) level.

*The value of t is indicated in parentheses.*

As for the moderating effect of Knd put forward by Hypothesis 5, this paper uses the OLS regression model to test and construct the interactive terms between them based on standardizing strategic sensitivity and Knd. The results show that the interactive terms have a significant positive impact on BMI (β = 0.45, *p* < 0.01), which indicates that Hypothesis 5 is verified.

### Endogenous Test

For the endogenous issues, we used the structural equation model to solve this problem as follows. Firstly, the structural equation model is constructed according to the research framework shown in Figure 1, and various fitting indexes of the model are calculated (CFI = 0.91, TLI = 0.90, RMSEA = 0.07, SRMR = 0.06). Secondly, the directions of Hypothesis 1a, Hypothesis 1b, and Hypothesis 1c are reversed, and the structural equation model is reconstructed, and the fitting coefficient of the new model is observed (CFI = 0.82, TLI = 0.81, RMSEA = 0.08, SRMR = 0.07). Thirdly, by comparing the fitting coefficient between the two models, it is found that the fitting coefficient of the original model is significantly better than that of the new model. Therefore, the results of this study are robust.

## Machine Learning: Decision Tree Algorithm Analysis

Machine learning abandons the traditional statistical method that relies on the hypothesis to verify and uses algorithms to analyze data, learn from them, and predict events. It is a unique measurement method that uses actual prediction accuracy as performance evaluation (Jordan and Mitchell, 2015). In this paper, the machine learning algorithm of the Decision tree is adopted through comparative analysis, model evaluation of training sets, and test sets of different learning models; a prediction model for BMI of new ventures is presented.

In this paper, configuration cognition, willingness cognition, Abc, strategic sensitivity, and Knd in entrepreneurial cognition are selected as input feature vectors of the conditional reasoning tree, which are denoted as $x_{i}^{v}$, and BMI of new ventures is denoted as *y*_*i*_, thus forming the original data set of machine learning model *D* = $\left\{x_{i}^{v},y_{i}\right\}{}_{i-1}^{n}$.

The method of information gain is selected as the division standard of the optimal attribute of decision tree learning, and the information entropy is defined as:


$$Ent\left(D\right)=-\sum_{k=1}^{\left|y\right|}P_{k}\log_{2}\left(P_{k}\right)$$

The information gain of sample attribute a is defined as:


$$Gain\left(D,a\right)=Ent\left(D\right)-\sum_{v=1}^{V}\frac{\left|D^{v}\right|}{\left|D\right|}Ent\left(D^{v}\right)$$

k stands for the sample category, _*P_k_*_ represents the proportion of the k sample in the sample set D, V represents the value range of the sample attribute a, v represents the branch node of the decision tree, and _|*D^v^*|/|*D*|_ represents the weight of a branch node.

**FIGURE 3:**
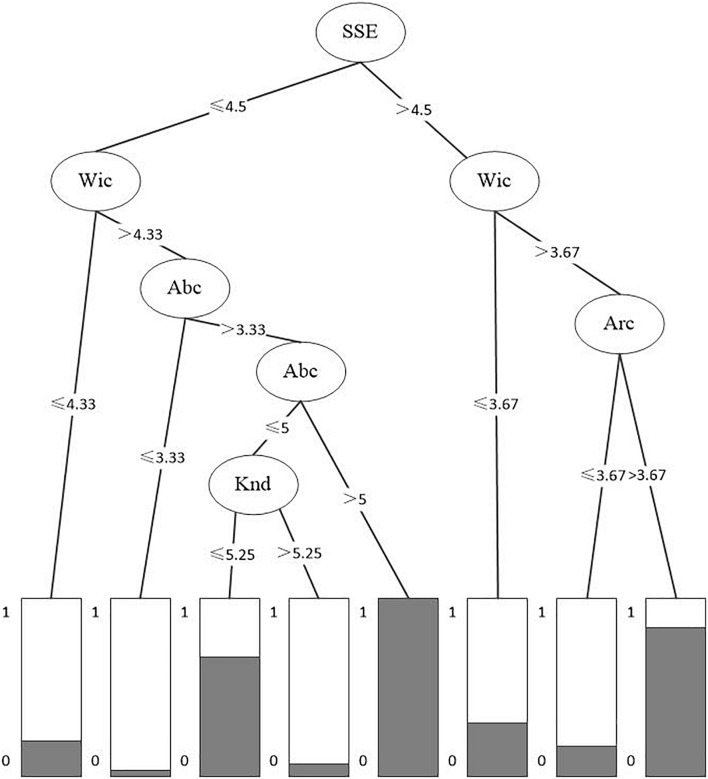
Decision tree prediction model results.

In this paper, the “hold-out” method is used to evaluate the generalization error of the machine learning model. Firstly, the original data set is divided into a training set and a test set according to the ratio of 7:3 by using the self-built function “split.data”; a prediction model is built based on the training set. Then, the prediction model is applied to the test set in the model evaluation stage. Based on installing and importing the “C5.0” package in R3.6.1 software, C5.0 algorithm is used to construct the prediction model, which takes the innovation of the business model of new ventures as the class label, and takes the cognition of entrepreneurial configuration, entrepreneurial willingness, entrepreneurial ability, strategic sensitivity, and Knd as the training attributes. The results of model training are shown in Figure 3.

It can be seen from the data shown in Figure 3 That there are eight forecasts in this decision tree; among which, there are three forecasting models that can lead to BMI.

In Model 1, the root node is strategic sensitivity (SSE > 4.5), the internal node is entrepreneurial willingness cognition (Wic > 3.67), and the leaf node is entrepreneurial configuration cognition (Arc > 3.67). Previous studies have pointed out that strategic sensitivity, as a capability combined with discovery-based entrepreneurial opportunities, emphasizes the continuous detection of various internal and external factors of enterprises, thus helping enterprises to produce innovative behaviors. From the analysis results of this paper, it can be seen that the influence of strategic sensitivity on BMI of new ventures also depends on other factors. Entrepreneurs with the high entrepreneurial cognitive structure are more likely to promote BMI of enterprises. Specifically, entrepreneurs with the high willingness cognition and configuration cognition can continuously transform strategic sensitivity into actual innovation behavior of enterprises through special knowledge structure and opportunity ability.

In Model 2, the root node is strategic sensitivity (SSE ≤ 4.5), the internal first-level node is entrepreneurial willingness cognition (Wic > 4.33), the internal second-level node is entrepreneurial Abc (Abc > 3.33), and the leaf node is entrepreneurial Abc (Abc > 5). It can be seen from the results of Model 2 that, when the strategic sensitivity is relatively low, in order to realize BMI, entrepreneurs need not only willingness cognition but also a high level of Abc so as to make favorable decisions for BMI through entrepreneurial experience involvement, entrepreneurial diagnosis, entrepreneurial situation cognition, and opportunity discovery.

In Model 3, the root node is strategic sensitivity (SSE ≤ 4.5), the internal first-level node is entrepreneurial willingness cognition (Wic > 4.33), the internal second-level node is entrepreneurial Abc (Abc > 3.33), the internal third-level node is entrepreneurial Abc (NL ≤ 5), and the leaf node is Knd (Knd ≤ 5.25). After comparing Model 3 with Model 2, it is found that, when the Abc of entrepreneurs is not high enough, new ventures need additional knowledge structure to realize BMI. The analysis results based on machine learning show that, on the one hand, Knd provides enough information structure and information analysis ability for new ventures and entrepreneurs. On the other hand, it makes new ventures face a dilemma when faced with massive information. Maintaining a reasonable level of Knd is helpful for new ventures to realize BMI.

The errors part of the prediction model shows that the model correctly classifies all cases except 22 of the 170 training cases, and the error rate is 12.9%. A total of 16 instances with a true value of 0 were erroneously classified as 1 (false positive), while 6 instances with a true value of 1 were erroneously classified as 0 (false negative). Furthermore, this paper evaluates the decision tree model by using the test data set, and the results are shown in Figure 3. Among the 72 test cases, the prediction model correctly predicts 61 test cases, and the test set shows that the accuracy of the model is 84.7%, which shows that the model has good prediction ability.

## Discussion

China is in a critical period of economic transformation as an important force to solve the employment problem and promote the economic development of China; new ventures have been facing the problem of a high-failure rate. How to help new ventures grow and strengthen in the fierce market competition has become the focus of current academic and practical circles. Based on social cognition theory, this paper constructs a multiple regression model to explore the influence of entrepreneurial cognition on BMI and analyzes the mediating role of strategic sensitivity and the moderating role of Knd. The empirical research results based on MLR show that three subdimensions of entrepreneurial cognition have a significant role in promoting BMI. Different subdimensions of entrepreneurial cognition are helpful to improve the level of strategic sensitivity. Strategic sensitivity plays a partial mediated role between entrepreneurial configuration cognition, entrepreneurial willingness cognition, entrepreneurial Abc, and BMI. The higher the level of Knd, the stronger the positive effect of strategic sensitivity on BMI.

The analysis of machine learning algorithm based on the decision tree shows that three prediction models can lead to BMI: the first model is strategic sensitivity, entrepreneurial willingness cognition, and entrepreneurial configuration cognition. The second model is strategic sensitivity, entrepreneurial willingness cognition, and entrepreneurial Abc. The third model is strategic sensitivity, entrepreneurial willingness cognition, entrepreneurial Abc, and Knd. It shows that there are many ways for new ventures to realize BMI.

### Theoretical Implications

The theoretical contribution of this study is mainly reflected in four aspects: First, the existing literature mainly pays attention to the antecedents of BMI from an organizational level, and the research on the mechanism of influencing BMI from the perspective of individual entrepreneurs is still relatively lacking. This paper combining with social cognitive theory discusses how new ventures with imperfect organizational structures and shortages of resources can realize BMI under the guidance of the cognition of entrepreneurs. The research conclusion not only deepens the relevant research on BMI of new ventures but also echoes Guo et al. (2013) that the research on BMI should pay attention to individual factors of entrepreneurs.

Second, although the existing studies have found the unique characteristics and abilities of entrepreneurs compared with non-entrepreneurs, entrepreneurs can inadvertently discover the characteristics and abilities of entrepreneurial opportunities without deliberately searching; the studies have not thoroughly explored the foundation of the characteristics and abilities. The research in this paper confirms the promotion of entrepreneurial cognition to strategic sensitivity and the mediated role of strategic sensitivity between entrepreneurial cognition and BMI. This paper not only clarifies the origin of the unique characteristics and abilities of entrepreneurs but also provides a new research perspective for analyzing the influence mechanism of entrepreneurial cognition on BMI, enriching the research results in the field of “situation-thinking behavior.”

Third, this paper finds and confirms that Knd plays a positive role in the relationship between strategic sensitivity and BMI. The boundary condition for strategic sensitivity to promote the efficiency of BMI lies in the rich level of Knd, which can help entrepreneurs find and judge whether market opportunities are profitable in information processing, external knowledge capture, and internal knowledge innovation, and help new ventures create new products and services that are more in line with customer needs. This further clarifies the important role of knowledge in the development of new ventures, and, at the same time, it also responds to the research of Larrañeta et al. (2012) that the heterogeneity and novelty of knowledge can promote the innovation of the business model.

Fourthly, this paper combines multiple linear regression model analysis with machine learning analysis, which not only verifies the research hypothesis of this paper through empirical analysis but also explores the prediction model of BMI by using the decision tree analysis algorithm of the machine learning method. It is found that strategic sensitivity is the necessary condition, leading to BMI, and its combination with different entrepreneurial cognitive subdimensions will have different effects, only by comprehensively considering the combined effects of multiple factors can the BMI be predicted and analyzed more effectively (Mai et al., 2021).

### Practical Implications

The research results of this paper have important practical implications. From the perspective of entrepreneurs, first, this paper verifies that entrepreneurial cognition at different subdimensions can significantly promote BMI. Therefore, entrepreneurs can carry out targeted training to enhance their different cognitive subdimensions. For example, they can participate in entrepreneurial cognitive training, build entrepreneurial cognitive network, and enhance entrepreneurial cognitive ability, and actively seek to combine their cognitive advantages with enterprise strategy to actively carry out BMI (Chen et al., 2018). Secondly, this paper verifies the important role of strategic sensitivity in the process of transforming entrepreneurial cognition into BMI; part of strategic sensitivity is the innate characteristics of entrepreneurs, and the other part is that entrepreneurs can acquire it through acquired training. Therefore, entrepreneurs should constantly accumulate previous experience, deeply understand the selected entrepreneurial industries, and accumulate industry information to improve their strategic sensitivity. Third, entrepreneurs need to continuously search and accumulate knowledge resources using attention allocation and information screening to improve the diversity level of resources within enterprises and make good reserves of knowledge resources for the implementation of BMI.

From the perspective of enterprises, new ventures should actively create conditions to give full play to the cognitive advantages of entrepreneurs. For example, they can innovate management systems, formulate institutional platforms for cultivating and improving entrepreneurial cognition, and provide reasonable institutional arrangements for the different cognitive dimensions of entrepreneurs, thus improving the conversion efficiency of “cognition behavior.” New ventures should constantly encourage employees to innovate and mobilize their enthusiasm for innovation. For example, Haier’s Maker Incubation Platform has stimulated a large number of employees to try BMI and achieved good results.

From the perspective of government, the government needs to provide a good entrepreneurial environment to promote the innovation and entrepreneurship of entrepreneurs. On the one hand, the government can improve the cooperation opportunities between universities and enterprises by providing conditions for university-enterprise docking, and cultivate entrepreneurial awareness of college students at different levels, consciously improve their strategic sensitivity, and thus cultivate potential outstanding entrepreneurs. On the other hand, the government should actively provide a favorable entrepreneurial environment for new ventures through policy support and financial support to encourage enterprises to actively innovate. For example, through the support of the government for new ventures, Shenzhen has formed an industrial model focusing on cultural entrepreneurship and high-tech industries, which has promoted the economic development of Shenzhen.

### Limitations and Future Research Directions

Although this study makes several critical contributions, it also has some limitations and further suggestions for entrepreneurial research. First, the measurement tools used in this paper mainly come from mature scales, which may fail to fully consider the entrepreneurial situation of China; future research can develop measurement tools in the entrepreneurial situation of China to obtain more accurate research conclusions. Second, in this paper, questionnaires are distributed in different regions to ensure the representativeness of the research, but the questionnaire recovery rate is low, which may lead to some limitations in the sample size; future research can consider increasing the sample size to ensure the popularization of the research results. Third, this paper adopts cross-sectional data for research and lacks to examine the cognition and innovation change process of entrepreneurs in vertical time series; dynamic data of entrepreneurship cognition and the BMI can be obtained through cross-stage tracking to explore the BMI comprehensively. Fourth, the factors affecting BMI are diverse, and the accuracy of the model predicted by machine learning algorithm needs to be improved. Therefore, the follow-up study can consider building a more accurate prediction model of BMI from a more comprehensive perspective to guide the practical activities more effectively.

## Data Availability Statement

The original contributions presented in the study are included in the article/supplementary material, further inquiries can be directed to the corresponding author/s.

## Ethics Statement

Ethical review and approval was not required for the study on human participants in accordance with the local legislation and institutional requirements. Written informed consent from the participants was not required to participate in this study in accordance with the national legislation and the institutional requirements.

## Author Contributions

JZ was responsible for idea generation, manuscript writing for a theoretical part, data collection, and data analysis. JY was responsible for idea generation and manuscript revision. HS was responsible for initial method part writing. YL was responsible for data analysis and manuscript revision. XL was responsible for manuscript revision. All the authors contributed to the article and approved the submitted version.

## Conflict of Interest

The authors declare that the research was conducted in the absence of any commercial or financial relationships that could be construed as a potential conflict of interest.

## Publisher’s Note

All claims expressed in this article are solely those of the authors and do not necessarily represent those of their affiliated organizations, or those of the publisher, the editors and the reviewers. Any product that may be evaluated in this article, or claim that may be made by its manufacturer, is not guaranteed or endorsed by the publisher.
